# Matrix Metalloproteinase-2 Impairs Homing of Intracoronary Delivered Mesenchymal Stem Cells in a Porcine Reperfused Myocardial Infarction: Comparison With Intramyocardial Cell Delivery

**DOI:** 10.3389/fbioe.2018.00035

**Published:** 2018-04-04

**Authors:** Katrin Zlabinger, Dominika Lukovic, Rayyan Hemetsberger, Alfred Gugerell, Johannes Winkler, Ljubica Mandic, Denise Traxler, Andreas Spannbauer, Susanne Wolbank, Gerald Zanoni, Christoph Kaun, Aniko Posa, Andrea Gyenes, Zsolt Petrasi, Örs Petnehazy, Imre Repa, Renate Hofer-Warbinek, Rainer de Martin, Florian Gruber, Silvia Charwat, Kurt Huber, Noemi Pavo, Imre J. Pavo, Noemi Nyolczas, Dara L. Kraitchman, Mariann Gyöngyösi

**Affiliations:** ^1^Department of Cardiology, Medical University of Vienna, Vienna, Austria; ^2^Ludwig Boltzmann Institute for Clinical and Experimental Traumatology/AUVA Research Center Austrian Cluster for Tissue Regeneration, Vienna, Austria; ^3^Institute of Biophysics, Biological Research Center, Szeged, Hungary; ^4^Institute of Diagnostics and Radiation Oncology, University of Kaposvar, Kaposvar, Hungary; ^5^Department of Biomolecular Medicine and Pharmacology, Institute of Vascular Biology and Thrombosis Research, Medical University of Vienna, Vienna, Austria; ^6^Department of Dermatology, Medical University of Vienna, Vienna, Austria; ^7^3rd Department of Medicine (Cardiology and Emergency Medicine), Wilhelminenhospital, Vienna, Austria; ^8^Russell H. Morgan Department of Radiology and Radiological Science, School of Medicine, The Johns Hopkins University, Baltimore, MD, United States

**Keywords:** mesenchymal stem cells, translational research, cell delivery, oxidative stress, homing, intracoronary, intramyocardial, ischemic injured heart tissue

## Abstract

**Background:**

Intracoronary (IC) injection of mesenchymal stem cells (MSCs) results in a prompt decrease of absolute myocardial blood flow (AMF) with late and incomplete recovery of myocardial tissue perfusion. Here, we investigated the effect of decreased AMF on oxidative stress marker matrix metalloproteinase-2 (MMP-2) and its influence on the fate and homing and paracrine character of MSCs after IC or intramyocardial cell delivery in a closed-chest reperfused myocardial infarction (MI) model in pigs.

**Methods:**

Porcine MSCs were transiently transfected with Ad-Luc and Ad-green fluorescent protein (GFP). One week after MI, the GFP-Luc-MSCs were injected either IC (group IC, 11.00 ± 1.07 × 10^6^) or intramyocardially (group IM, 9.88 ± 1.44 × 10^6^). AMF was measured before, immediately after, and 24 h post GFP-Luc-MSC delivery. *In vitro* bioluminescence signal was used to identify tissue samples containing GFP-Luc-MSCs. Myocardial tissue MMP-2 and CXCR4 receptor expression (index of homing signal) were measured in bioluminescence positive and negative infarcted and border, and non-ischemic myocardial areas 1-day post cell transfer. At 7-day follow-up, myocardial homing (cadherin, CXCR4, and stromal derived factor-1alpha) and angiogenic [fibroblast growth factor 2 (FGF2) and VEGF] were quantified by ELISA of homogenized myocardial tissues from the bioluminescence positive and negative infarcted and border, and non-ischemic myocardium. Biodistribution of the implanted cells was quantified by using Luciferase assay and confirmed by fluorescence immunochemistry. Global left ventricular ejection fraction (LVEF) was measured at baseline and 1-month post cell therapy using magnet resonance image.

**Results:**

AMF decreased immediately after IC cell delivery, while no change in tissue perfusion was found in the IM group (42.6 ± 11.7 vs. 56.9 ± 16.7 ml/min, *p* = 0.018). IC delivery led to a significant increase in myocardial MMP-2 64 kD expression (448 ± 88 vs. 315 ± 54 intensity × mm^2^, *p* = 0.021), and decreased expression of CXCR4 (592 ± 50 vs. 714 ± 54 pg/tissue/ml, *p* = 0.006), with significant exponential decay between MMP-2 and CXCR4 (*r* = 0.679, *p* < 0.001). FGF2 and VEGF of the bioluminescence infarcted and border zone of homogenized tissues were significantly elevated in the IM goups as compared to IC group. LVEF increase was significantly higher in IM group (0.8 ± 8.4 vs 5.3 ± 5.2%, *p* = 0.046) at the 1-month follow up.

**Conclusion:**

Intracoronary stem cell delivery decreased AMF, with consequent increase in myocardial expression of MMP-2 and reduced CXCR4 expression with lower level of myocardial homing and angiogenic factor release as compared to IM cell delivery.

## Introduction

Since the endogenous cardiac progenitor cells are unable to restore the cardiac function in toto, tissue regeneration of the heart by delivering exogenous reparative cells has been intensively studied. Clinical stem cell therapy was assumed to have beneficial effects on the regeneration of the ischemic injured myocardium; nevertheless, recent clinical studies and meta-analyses revealed that intracoronary (IC) administration of bone marrow-origin mononuclear cells has no impact on left ventricular function and clinical outcome (Gyongyosi et al., 2015).

Mesenchymal stem cells (MSCs) and other progenitor or stem cells secrete various types of cytokines, growth factors, and chemokines under *in vitro* conditions that play an important role in cardiac remodeling, angiogenesis, apoptosis, and survival (Gnecchi et al., 2008). The regenerative mechanism might be attributed to secretion of paracrine factors (Thum et al., 2005); therefore, MSCs are increasingly used in human clinical trials (Roura et al., 2017).

Myocardial engraftment kinetics of cardiac transplanted stem cells play a highly relevant role in the regeneration of cardiac tissue. Impaired homing of the cells may be one reason of the failure of stem cell therapy (Chavakis et al., 2008; Penn and Mangi, 2008; Schoenhard and Hatzopoulos, 2010; Wollert and Drexler, 2010). Our group as well as others have previously reported that due to possible cell sludge formation and microvascular obstruction, experimental IC injection of MSCs results in prompt decrease of absolute myocardial blood flow (AMF) with late and incomplete recovery of tissue perfusion (Vulliet et al., 2004; Gyongyosi et al., 2011). This leads to an increase in intraluminal pressure, inhibiting cell passage distal to the ischemic injured area, and the development of acute local ischemia with enhanced oxidative stress, hampering accumulation, and homing of the cells in the peri-infarcted area (Vulliet et al., 2004; Gyongyosi et al., 2010a,b, 2011).

The stromal cell-derived factor (SDF)-1/chemokine (C-X-C motif) receptor 4 [(SDF)-1/CXCR4] axis is one of the most important factors in stem/progenitor cell homing, chemotaxis, engraftment, and retention into ischemic tissue (Wojakowski et al., 2004). Enhanced expression of matrix metalloproteinase 2 (MMP-2) due to ischemia-induced oxidative stress has been shown to interrupt the SDF/CXCR4 axis due to SDF-1alpha proteolysis, thereby limiting homing (Giricz et al., 2006; Segers et al., 2007; Rota et al., 2008; Lukovic et al., 2016). We have previously demonstrated *in vitro* that MMP-2 directly inhibited the SDF-1 alpha induced migration of CD34+ cells toward cardiomyocytes (Lukovic et al., 2016). The immediate activation of the MMP-2 during ischemia induces local inflammation and degradation of several cellular components *via* NF-kB and NFAT stress signaling pathways. Proteolytic fragments of MMP-2 provoke also autoimmune responses, leading to progressive cardiomyopathy and myocyte contractile dysfunction. MMP-2 plays also a role in pathological processes turning the reversible ischemic events to irreversible injury, contributing to development of heart failure (DeCoux et al., 2014).

In the present experiment, we have investigated the association between decreased myocardial blood flow and the acute oxidative stress marker MMP-2, and the effect of increased MMP-2 on the homing, biodistribution, and paracrine effect of the cardiac delivered MSCs, in the pig closed-chest, reperfused myocardial infarction (MI) model in a side-by-side comparison of IC and intramyocardial delivery modes.

## Materials and Methods

### Preparation and Transfection of MSC

Bone marrow (100 ml) of farm pigs was harvested from the iliac crest and stored at 4°C (Baxter bag, Baxter Healthcare, Ltd., Thetford, Norfolk, UK). The MSC were selected using Ficoll–Paque (Amersham Biosciences), cultured and transfected as described previously (Gyongyosi et al., 2008). Briefly, buffy coats were plated at 50,000 cells/cm^2^ in alpha MEM medium without nucleotides, containing 10% fetal calf serum (FCS), 2 mM l-glutamine, penicillin/streptomycin supplemented with 1 ng/ml fibroblast growth factor 2 (FGF2). The prepared cells were negative for CD45 (Bio-Rad Laboratories, Hercules, CA, USA), CD34 (Thermo Fisher Scientific, Waltham, MA, USA), and positive for CD44, CD90, and CD29 (all EXBIO Praha, Vestec, Czech Republic) expression (see Figure S1 in Supplementary Material).

After reaching the fourth passage, the cultured MSCs were transfected with the combination of Ad-CMV-Luc and Ad-CMV-green fluorescent protein (GFP) (Vector Biolabs, Philadelphia, PA, USA). The AdV was then removed and fresh medium was added. Transfection efficiency was determined by flow cytometry. The viability of the GFP-Luc-MSC was assessed prior to cardiac delivery using trypan blue (Sigma-Aldrich, Saint Louis, MO, USA) staining. A proliferation assay was performed 1, 2, 4, and 8 days after seeding, using an EZ4U kit (Biomedica, Vienna, Austria) according to manufacturer’s protocol.

### Induction of AMI in Female Pigs

All animal studies were approved by the local Experimental Animal Care Committee of University of Kaposvar, Hungary where the experiments were performed (EC 246/002/SOM2006, MAB-28-2005). The experiments conform to the “Position of the American Heart Association on Research.”

Closed-chest, acute reperfused MI was induced in 39 female domestic pigs (23 ± 3 kg) by percutaneous catheter-based balloon occlusion of the left anterior descending coronary artery (LAD), as described previously (Gyongyosi et al., 2011). Briefly, after direct puncture of the right femoral artery and administration of 200 IU/kg of heparin, a 90-min percutaneous balloon (3.0 mm in diameter × 15 mm long, Maverick, Boston Scientific Corp., Natick, MA, USA) occlusion (5 atm) of the mid LAD, beyond the origin of the second major diagonal branch was performed. The complete occlusion of the artery distal to the balloon was confirmed by angiography. After a 90-min LAD occlusion, the balloon was deflated slowly to allow reperfusion. The puncture site was then closed with Angioseal (St. Jude Medical, St. Paul, MN, USA), and the pigs were allowed to recover.

**Figure 1:**
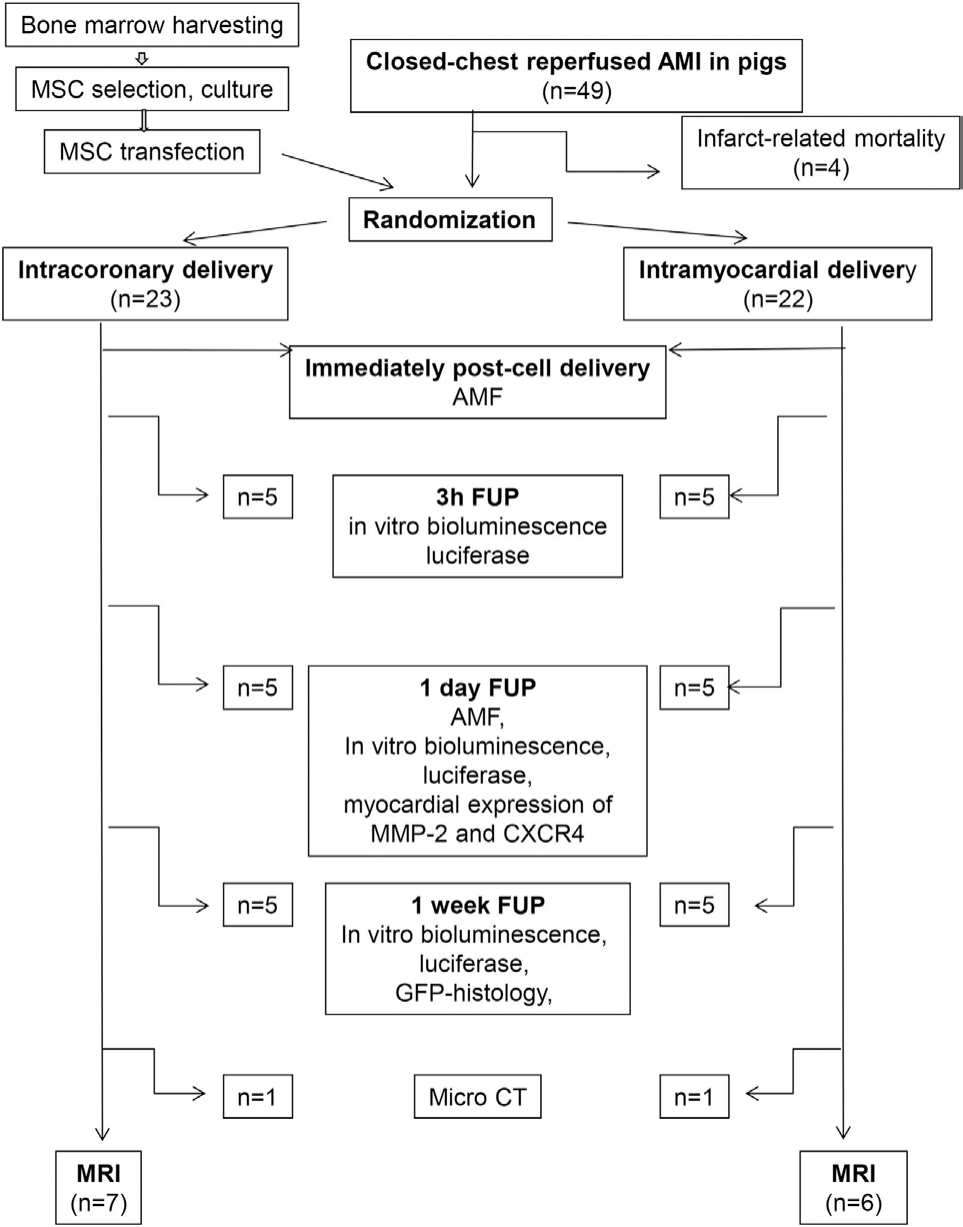
Study design of the experiment. MSC, mesenchymal stem cell; AMF, absolute myocardial blood flow; FUP, follow-up; MMP-2, matrix metalloproteinase; GFP, green fluorescent protein; micro CT, micro computed tomography; MRI, magnet resonance image.

One-week (8 ± 2 days) post-MI, the pigs were randomized to receive either IC infusion or 3D NOGA-guided percutaneous intramyocardial injections of the GFP-Luc-MSC. The study design with different follow-ups (FUPs) is shown in Figure 1.

### Cardiac Delivery of GFP-Luc-MSCs

For intramyocardial cell administration (group IM), an 8F sheath (Terumo Medical Corporation) was placed in the left femoral artery, and a diagnostic NOGA catheter (Cordis, Johnson & Johnson, Miami Lakes, FL, USA) was advanced through the aortic valve into the left ventricle. Detailed descriptions of the endocardial mapping, injection system components, and principles have been described elsewhere (Ben-Haim et al., 1996; Gepstein et al., 1997). Briefly, the left ventricular endocardial surface was mapped by measuring the local electrical activity and displayed as 3D color-coded map with clear distinction of the viable and nonviable myocardial areas and the border zone of infarction. After replacement of the diagnostic mapping catheter through the Myostar (Cordis, Johnson & Johnson, Miami Lakes, FL, USA) injection catheter, the GFP-Luc-MSCs were injected into the peri-infarct myocardium at 12–13 sites. The injections (0.3 ml cell suspension each) were given slowly (40–45 s) to areas with a unipolar voltage above 6 mV, based on the quality control criteria (Ben-Haim et al., 1996; Gepstein et al., 1997).

For IC cell administration (group IC), a guiding catheter (Medtronic Minneapolis, MN, USA) was introduced into the ostium of the LAD. A Sprinter^®^ Over-the-Wire Semicompliant Balloon Catheter (Medtronic, Minneapolis, MN, USA) was introduced into the LAD, distal to the second diagonal branch over a guide wire, and a heparin diluted cell suspension (up to 10 ml) of male GFP-Luc-MSCs was slowly injected with stop-flow technique over about 15 min. The patency of the target vessel following the injection was confirmed *via* angiography.

### Measurement of AMF

Absolute myocardial blood flow was measured before, immediately after, and at 24 h post-IC and intramyocardial (*n* = 5 of each group) delivery of the GFP-Luc-MSCs.

The measurement of AMF has been described previously (Aarnoudse et al., 2007). Briefly, a 0.014″ pressure wire (Radi Medical Systems, Uppsala, Sweden) was positioned in the distal LAD. A specially designed infusion catheter (Occam Inc., Eindhoven, Netherlands) was advanced over the pressure wire until its tip was just proximal to the flow probe. The infusion catheter was connected to an infusion pump (Stellant D CT Injector, Medrad, Warrendale, PA, USA) and saline (12 ml/min) was infused through this infusion catheter. Maximum hyperemia was induced by continuous intravenous administration of 140 µg/kg/min adenosine. The infusion of saline (20°C) was started and upon reaching a steady-state continuous infusion, the decrease of blood temperature (T) was measured. Volumetric blood flow in the LAD was calculated according to predefined formula.

### Tracking of the Cardiac-Delivered GFP-Luc-MSCs: *In Vitro* Bioluminescence Imaging, Luciferase Assay, and Immunohistochemistry

In order to make the injected cells visible in the heart and remote organs, *in vitro* bioluminescence images were performed, as described previously (Gyongyosi et al., 2010a, 2011). Briefly, the hearts and different organs were harvested immediately after euthanasia, cut in 1 cm thick slices, then placed in 20 ml of ATP (200 µM) and luciferin (3 mg/ml) in Ringer solution for 5 min. Photon emission of metabolically active, Luc-expressing cells, close to the tissue surface were captured with a charged-coupled device camera system (IVIS, Xenogen Corp.). Tissue pieces (1–2 mg/each sample) positive for bioluminescence (infarcted and border zona), as well as bioluminescence negative infarcted and border zone and non-ischemic myocardium (remote posterior wall) were cut and prepared for quantitative luciferase measurements and immunohistochemistry.

For measurements of luciferase activity, tissue samples were collected from the infarcted and border zone (bioluminescence positive and negative), and non-ischemic myocardium and different organs of the animals (pericardium, pleura, lung, mediastinal lymphatic nodes, liver, spleen, kidney, skin and bone marrow from the iliac crest) at 3, 24 h, and 7-days post cell delivery. The tissues were processed for measurement of luciferase activity as described previously (Gyongyosi et al., 2011). Luciferase was determined using components of the Dual-Luciferase Reporter Assay System (Promega, Madison, WI, USA) (Gyongyosi et al., 2010b). Results were given in relative light units (RLU) per microgram protein.

Bioluminescence positive tissue samples were fixed in 4% buffered paraformaldehyde and embedded in paraffin for visualization of the GFP+ cells in different organs. Cellular nuclei were counterstained with Hoechst.

### Measurement of Oxidative Stress by Cardiac Tissue MMP-2 Activity

To quantify the stem cell infusion-induced increase in oxidative stress, cardiac tissue MMP-2 activities of five myocardial areas (bioluminescence positive infarcted and border zone, bioluminescence negative infarcted and border zone, and non-ischemic myocardium) were measured by zymographic analysis 24 h post cell delivery. Briefly, polyacrylamide gels (8%) were copolymerized with gelatin (2 mg/ml, Sigma-Aldrich), and a constant amount of myocardial tissue homogenate was loaded in each lane. Following electrophoreses at 150 V for 1.5 h, gels were washed with 2.5% Triton X-100 for 3 × 15 min and incubated for 24–48 h at 37°C in incubation buffer (50 mM Tris–HCl, 150 mM NaCl, 5 mM CaCl2, and 0.05% NaN3, pH 7.4). Gels were then stained with 0.05% Coomassie Brilliant Blue (G-250; Sigma-Aldrich) in a mixture of methanol/acetic acid/water (2.5:1:6.5, v/v) and destained in aqueous 4% methanol/8% acetic acid. Gelatinolytic activities were detected as transparent bands against the dark–blue background. Zymograms were digitally scanned, and band intensities were quantified using Quantity One software (Bio-Rad, Hercules, CA, USA).

Values of 24 h follow-up (FUP) AMF of both groups and MMP-2 64 kD activities of the bioluminescence infarcted area of both IC and IM groups were pooled to investigate the direct effect of decreased AMF on increase in MMP-2 64 kD.

### Homing and Paracrine Effect of Cardiac-Delivered GFP-Luc-MSCs

At the 7 days FUP, bioluminescence positive and negative infarcted and infarct-border cardiac tissue samples, as well as samples from the remote non-ischemic myocardium were either processed to immunohistochemistry, or homogenized for tissue ELISA assays.

For immunohistochemistry, myocardial areas were fixed in 4% buffered paraformaldehyde and embedded in paraffin. To investigate the homing of the injected GFP-Luc-MSCs, myocardial expression of cadherin (Abcam, Cambridge, UK), and for angiogenic factors, vascular endothelial growth factor (VEGF) (Cusabio, Cologne, Germany) and FGF2 (Lifespan, Seattle, WA, USA) were displayed by immunofluorescence staining. The following secondary antibodies were used: Dylight 488-conjugated Donkey Anti-Goat IgG (705-485-003), Dylight 549-conjugated Goat Anti-Rabbit IgG (115-505-003), Dylight 549-conjugated Goat Anti-Mouse IgG (115-505-003), Dylight 488-conjugated Donkey Anti-Sheep IgG (713-485-003). Fluorescent images were acquired by an Olympus Provis AX 70 microscope (New Hyde Park, NY, USA).

To quantify the myocardial expression of homing and angiogenic signals, porcine CXCR4 (USCN Life Science, Wuhan, China), SDF-1alpha (Neoscientific, Germany), FGF2 (Neoscientific, Germany), VEGF (Neoscientific, Germany), and cadherin (MyBiosource Inc, San Diego, CA, USA) ELISAs were performed.

Additionally, in order to prove the direct correlation between increased MMP-2 activity and CXCR4 expression, bioluminescence positive infarcted and border areas were also homogenized and CXCR4 ELISA was performed at the 24 h FUP.

### Effect of Cardiac-Delivered GFP-Luc-MSCs on Coronary Microvascularization and Global Cardiac Function

Cardiac magnet resonance image (MRI) was performed using a 1.5-T clinical scanner (Avanto, Siemens, Erlangen, Germany) using a phased array surface coil and a vector ECG system, 1 ± 1 day before, and at the 4-week FUP. Cine MR images were acquired using ECG-gated, steady-state free precession cine MRI in short and long-axis views of the heart (1.2 ms echo time (TE), 40 ms repetition time (TR), 50° flip angle, 300 mm field-of-view, 8 mm slice thickness, and 256 × 256 image matrix). Sixteen short-axis images were acquired by ECG-gated, saturation-recovery true fast imaging with steady state precession (FISP) sequences. Following injection of 0.2 mmol/kg of contrast media, delayed enhancement images were acquired using an inversion recovery-prepared, gradient-echo sequence. Short-axis and long-axis views were obtained 10–15 min after gadolinium injection. Images were analyzed using the Mass 6.1.6 software (Medis, The Netherlands) to calculate end-diastolic and end-systolic volumes and global LV ejection fraction. The LV and infarcted myocardial mass were determined from the cine and delayed enhancement MR images, respectively. The infarct size was expressed relative to LV mass.

To determine microvascularization, micro-computer tomography (MicroCT) of GFP-Luc-MSC-treated infarcted hearts was performed in one animal from each treatment group. The pig hearts were removed and immersed in 4°C Krebs’ solution containing heparin and then prepared for microCT to allow quantification of the images of the infarcted area as described previously (Mondy et al., 2009). The coronary arteries were then immediately perfused with an isoosmotic, rinsing solution at 100 mmHg until the fluid ran clearly through the cardiac veins. The heart was placed in a saline bath and Tensol 70 was perfused through the major coronary arteries at 10 ml/h and at a pressure of 100 mmHg until the solution hardened. The cast preparation was completed within 2 h postmortem. The heart was then kept in a water bath at 50°C for 1 day to complete the polymerization process. The myocardial tissue was macerated with 7.5% KOH. The heart images were then captured using a VivaCt75 von Scanco Medical Basserdorf (50 kV, 1 s integration time, 900 projections, 1,024 × 1,024 a-Si flat panel detector, conebeam reconstruction) with a resolution of 80 µm.

### Statistics

For semiquantitative determination of luciferase, five samples of each organ and bioluminescence positive (infarct treated) and negative (infarct non-treated), as well as non-ischemic myocardium were measured and the mean values were calculated. Continuous parameters of the groups were compared by using ANOVA supplemented with unpaired *t*-test. Correlation between predefined parameters, such as decrease in AMF with increase in MMP-2 64 kD activity, or MMP-2 64 kD activity with myocardial expression of CXCR4 from the pooled bioluminescence positive infarcted and border area were calculated. In order to prove the direct inhibitory effect of increased MMP-2 64 kD on homing of GFP-Luc-MSCs, the 24 h follow-up data of Luciferase activity of the infarcted and non-infarcted areas were pooled and correlated with MMP-2 64 kD values.

Significance was considered if *p* < 0.05. All statistical tests were performed by using SPSS Mac V24.

## Results

### Cell Delivery

Proliferation assay indicated a temporary significant decrease in proliferation capacity of the transfected MSC as compared with the native MSC 96 h post-transfection (Figure S2 in Supplementary Material).

A total of 9.88 ± 1.44 × 10^6^ GFP-Luc-MSCs (12 ± 2 injection sites, 4.0 ± 0.2 ml cell suspension) was injected intramyocardially in Group IM, while 11.00 ± 1.07 × 10^6^ GFP-Luc-MSCs were injected IC in Group IC (12 ± 2 ml cell suspension).

**Figure 2:**
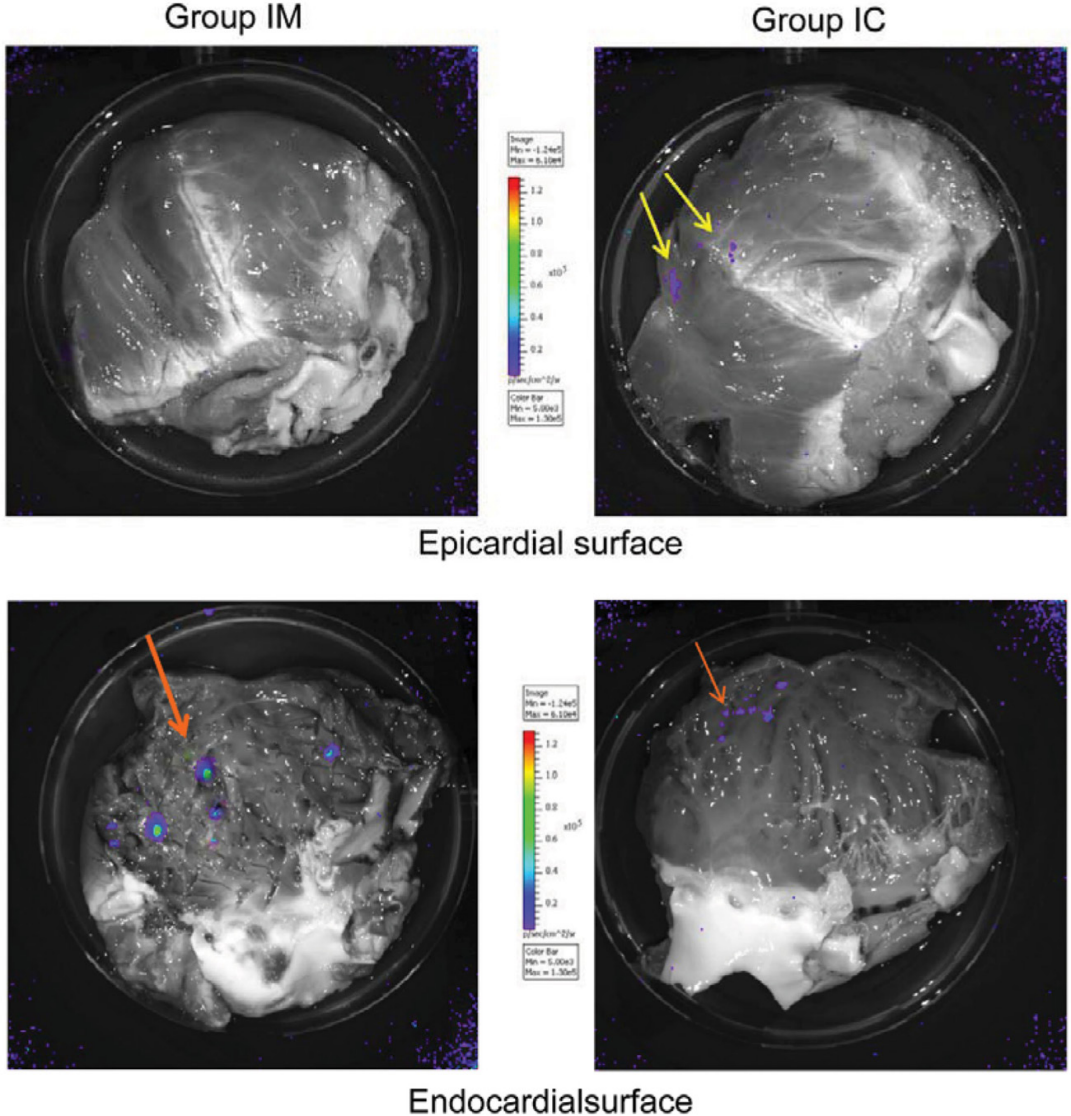
*In vitro* bioluminescence of pig hearts with intracoronary (IC) or intramyocardial delivery of green fluorescent protein (GFP)-Luc-mesenchymal stem cells (MSCs). *In vitro* bioluminescence images of Luc-transfected porcine MSC show epicardial perivascular location of cells after IC injections (upper right) in contrast to intramyocardial delivery (upper left). Highly positive endocardial punctual signals 24 h after intramyocardial GFP-Luc-MSCs delivery (bottom left). Weak confluent signal of GFP-Luc-MSCs on the endocardial surface of the heart after IC delivery (bottom right).

*In vitro* bioluminescence of the hearts showed less accumulation of the bioluminescence positive (Luc-containing) MSCs in the endocardial surface 24 h post IC delivery, as compared with intramyocardial application (Figure 2).

### Myocardial Blood Flow and Procedural Ischemia-Induced Changes in MMP-2 Expression

Absolute myocardial blood flow decreased immediately after IC delivery while no significant change in tissue perfusion could be detected using the percutaneous intramyocardial delivery mode (Table 1).

**Table 1:** Absolute myocardial blood flow before and after GFP-Luc-MSC intracoronary (IC) or percutaneous intramyocardial delivery.

Absolute myocardial blood flow (ml/min)	Intramyocardial group (*n* = 5)	IC group (*n* = 5)	*p-*Value
Pre GFP-Luc-MSC delivery	59.5 ± 11.3	59.9 ± 13.9	n.s.
Immediately after GFP-Luc-MSC delivery	56.9 ± 16.7	42.6 ± 11.7	0.018
24 h post GFP-Luc-MSC delivery	58.9 ± 8.7	45.5 ± 5.2	0.017

*GFP, green fluorescent protein; Luc, luciferase; MSC, mesenchymal stem cell*.

**Figure 3:**
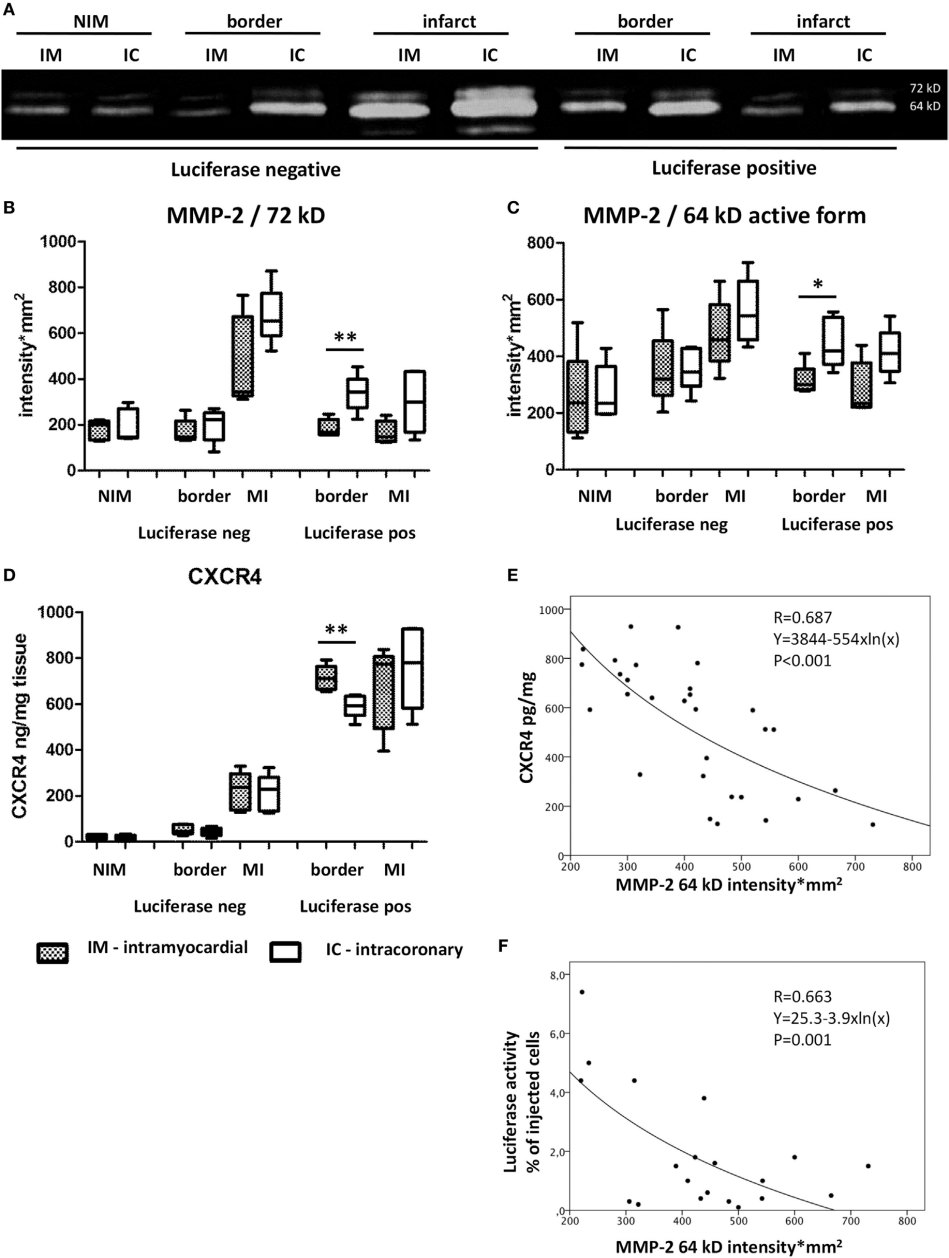
Oxidative stress and homing signals of the myocardium 24 h post acute myocardial infarction. Zymography **(A)** and statistical results **(B,C)** of matrix metalloproteinase-2 (MMP-2), 72 kD and its active form 64 kD expression of the myocardium 1 day after intramyocardial or IC green fluorescent protein (GFP)-Luc-mesenchymal stem cell (MSCs) cell delivery in different location. IM, intramyocardial delivery; IC, intracoronary delivery; NIM, non-ischemic myocardium (remote posterior wall), border, border zone of infarction; MI, infarcted area; **(D)** CXCR4 expression in the myocardial tissues 24 h after cell treatment. **(E)** Exponential decay between MMP-2 and CXCR4. **(F)** Logarithmic correlation between MMP-2 64 kD and luciferase activity (index of number of GFP-Luc-MSCs).

Parallel to the decrease of coronary blood flow in the IC group, myocardial expression of MMP-2 was higher in bioluminescence-positive border zones of the infarction as compared to the IM group (MMP-2 72 kD: 338 ± 81 vs 185 ± 38 intensity × mm^2^, *p* = 0.005; and MMP-2 64 kD: 448 ± 88 vs 315 ± 54 intensity × mm^2^, *p* = 0.006), suggesting the increase in acute oxidative stress due to cell slugging in the microvasculature (Figure 3).

In parallel, border zone tissue expression of CXCR4 was significantly lower in the IC delivery group as compared with the IM group (592 ± 50 vs 714 ± 54 pg/mg, *p* = 0.006), indicating decreased level of homing signal (Figure 3).

A significant, linear negative correlation was found between the AMF and MMP-2 64 kD myocardial expression (bioluminescence positive infarcted area, pooled data of both groups) (*r* = −0.838, *p* < 0.01, *y* = 622 − 5.3×) at the 24 h FUP indicating a direct, unfavorable influence of microvascular obstruction on homing signal.

The significant exponential decay of myocardial CXCR4 expression in relation to MMP-2 64 kD intensity in the pooled bioluminescence positive infarcted and border zone proved the direct anti-homing effect of increased MMP-2 activity (Figure 3). Additionally, a significant negative logarithmic correlation was found between the MMP-2 64 kD and measured Luciferase activity (number of GFP-Luc-MSCs) in the infarcted treated and infarcted non-treated myocardium (Figure 3). Pooling the MMP-2 64 kD and Luciferase data only of the infarcted treated (injected) area, the coefficient of variation was *r* = 0.729, with a *p* value of 0.017 (Figure S3 in Supplementary Material).

### Expression of Homing Factors in the Myocardium: Differences Between the IC and Intramyocardial Delivery Modes

Trend to higher levels of tissue expressions of homing signals cadherin and SDF-1alpha were seen both in ELISA and immunohistochemistry in the bioluminescence positive areas, supposing that the majority of the secreted paracrine factors were originated from the injected GFP-Luc-MSCs.

**Figure 4:**
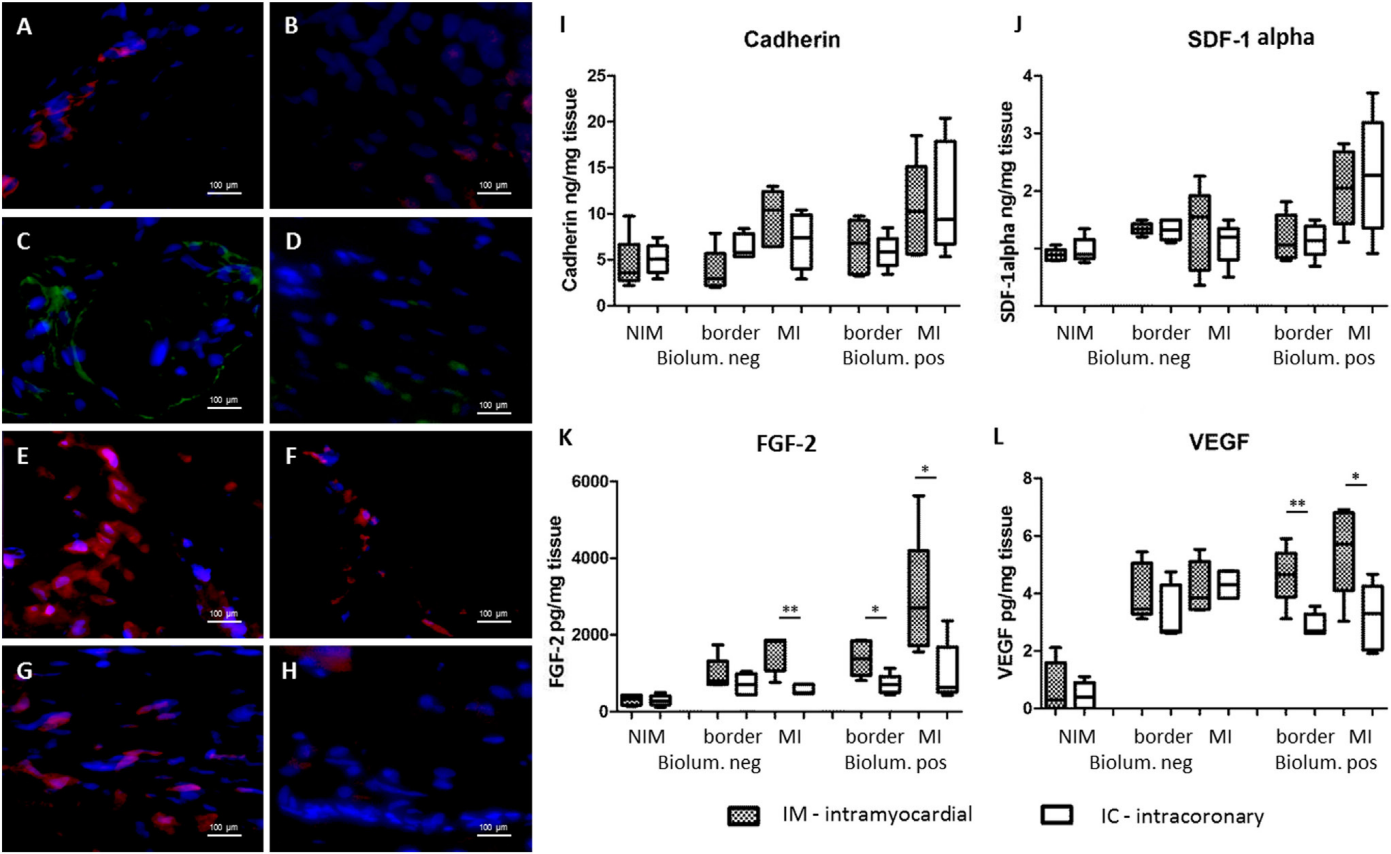
Expression of homing and angiogenic signals of the myocardium 7 days after cardiac transfer of green fluorescent protein (GFP)-Luc-mesenchymal stem cell (MSCs). Fluorescent immunohistochemistry of the bioluminescence positive myocardial areas 7 days after intramyocardial [left panel **(A,C,E,G)**] or intracoronary [right panel **(B,D,F,H)**] GFP-Luc-MSCs delivery shows increased expression of homing signals cadherin **(A,B)**, and angiogenic factors fibroblast growth factor 2 (FGF2) **(C,D)** and vascular endothelial growth factor (VEGF) **(E,F)** in group IM. Infarct area border zone **(G,H)** exhibited higher number of myocardial cells and higher level of VEGF expression in group IM **(G)**. Hoechst staining of the nuclei, 40× magnification. Expression of homing signals cadherin **(I)**, stromal-derived factor-1alpha **(J)**, and angiogenic factors FGF2 **(K)** and VEGF **(L)**.

At the 7-day FUP, significantly lower CXCR4 was found in the bioluminescence positive infarcted myocardium in the IC group, as compared with the IM group (129 ± 110 vs 411 ± 213 pg/mg, *p* = 0.03) (Figure 4), where the cells were injected. Myocardial levels of the angiogenic factors FGF2 were significantly decreased in the IC group in the bioluminescence positive border (710 ± 259 vs 1,390 ± 460 pg/mg, *p* = 0.02) and infarcted areas (1,002 ± 791 vs 2,903 ± 1,609 pg/mg, *p* = 0.045), but also in the bioluminescence negative infarct area (571 ± 127 vs 1,538 ± 479 pg/mg, p = 0.002). In parallel, lower VEGF level was detected in the bioluminescence infarcted (3.17 ± 1.15 vs 5.5 ± 1.6 pg/mg, *p* = 0.027) and border zone (2.89 ± 0.4 vs 4.64 ± 1.0 pg/mg, *p* = 0.007) in IC group vs IM group (Figure 4). Fluorescence immunochemistry confirmed the ELISA findings (Figure 4).

### Biodistribution of GFP-Luc-MSCs in the Heart and Remote Organs

**Figure 5:**
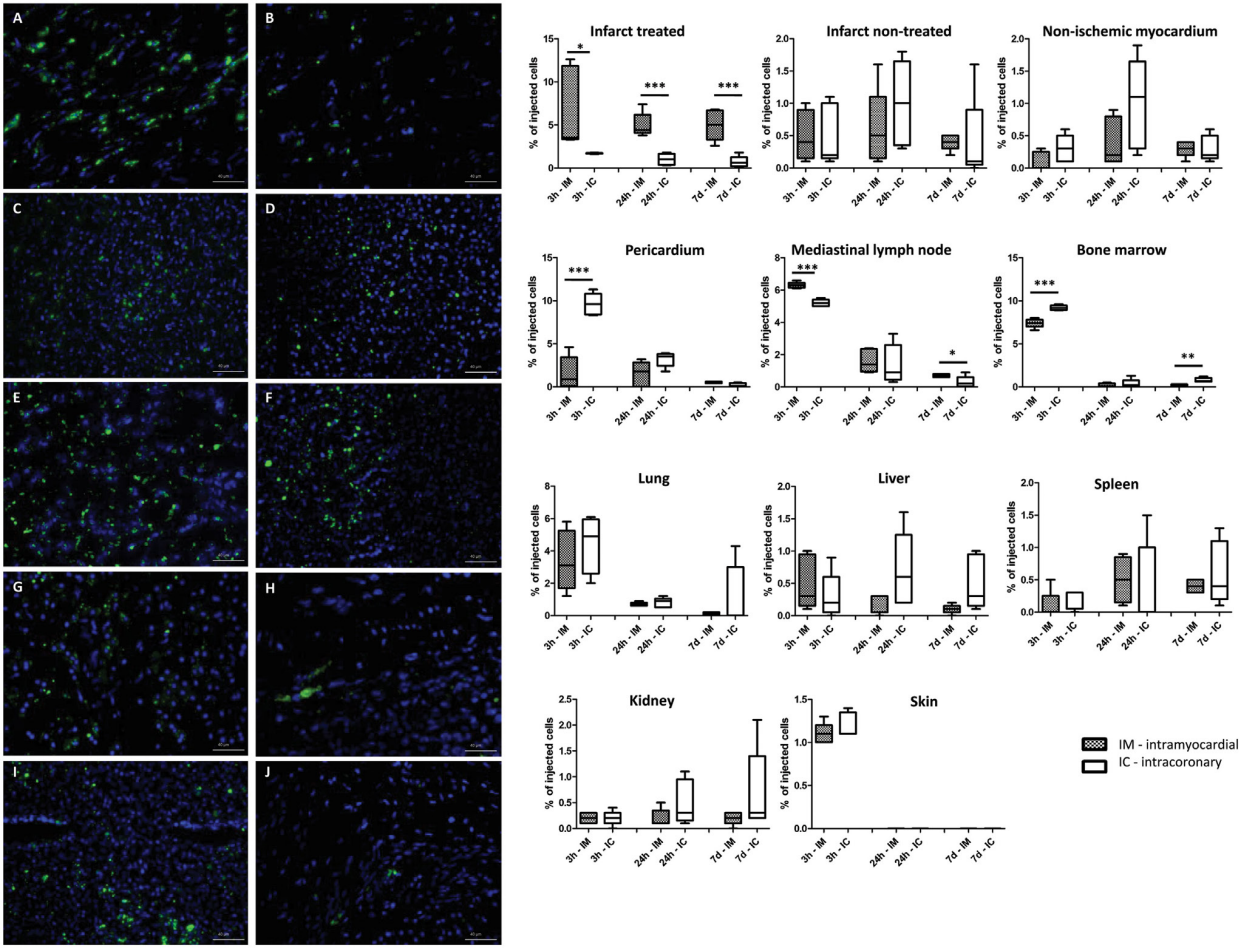
Time-dependent biodistribution of the intramyocardial and intracoronary (IC) delivered green fluorescent protein (GFP)-Luc-mesenchymal stem cell (MSCs). GFP+ positive cells in the infarcted heart tissue **(A,B)**, lung **(C,D)**, mediastinal lymph node **(E,F)**, liver **(G,H)**, and spleen **(I,J)** 7 days after intramyocardial (left panel, group IM) or IC (right panel, group IC) delivery of GFP-Luc-MSCs. Time response of luciferase activity is shown in the graphs on the right side.

Figure 5 shows the time-dependent accumulation of the injected cells in the heart and remote organs, expressed as the percentage of the originally injected GFP-Luc-MSCs. In accordance with the higher level of CXCR4 homing signal expression of the myocardium, the highest luciferase activity was found in the intramyocardially injected site 3 h post delivery (6.9 ± 5.9%), while IC delivery led to less cell retention (1.7 ± 0.1%) (*p* = 0.041). At 24 h and 7 days FUP, the number of cardiac amount of GFP-Luc-MSCs decreased, but the intramyocardial retention of the cells were still significantly higher in the IM group (Figure 5). In accordance with the rapid biodistribution of the cells, higher amount of GFP-Luc-MSCs could be found in bone marrow in the IC group 3 h post-delivery. Each remote organ contained a small amount of GFP-Luc-MSCs, even 1 week after cell delivery, with no difference between the groups.

Fluorescent microscopy confirmed the presence of GFP-positive cells in all sampled organs 1-week post GFP-Luc-MSCs delivery (Figure 5).

### Microvascularization and LV Function at 1-Month Follow-Up Post IC or Intramyocardial GFP-Luc-MSC Delivery

Magnet resonance image confirmed the significantly higher cardiac output and a significantly improved global ejection fraction in the IM group as compared to IC group (Table 2).

**Table 2:** Magnetic resonance image-derived left ventricular functional results 1 month after green fluorescent protein-Luc-MSC percutaneous intramyocardial or intracoronary (IC) delivery.

	Intramyocardial group (*n* = 5)	IC group (*n* = 5)	*p-*Value
3-days magnet resonance image (MRI)			
End-diastolic volume (ml)	65.8 ± 17.8	61.5 ± 20.6	n.s.
End-systolic volume (ml)	42.6 ± 13.1	37.6 ± 12.3	n.s.
Stroke volume (ml)	23.3 ± 5.2	23.8 ± 9.1	n.s.
Cardiac output (l/min)	1.6 ± 0.3	1.6 ± 0.3	n.s.
Ejection fraction (%)	36.2 ± 4.3	38.7 ± 5.2	n.s.
1-month follow-up MRI			
End-diastolic volume (ml)	78.9 ± 30.7	78.3 ± 33.6	n.s.
End-systolic volume (ml)	47.5 ± 22.7	47.5 ± 24.0	n.s.
Stroke volume (ml)	31.4 ± 9.3	30.9 ± 16.7	n.s.
Cardiac output (l/min)	2.3 ± 0.2	1.9 ± 0.5	0.049
Ejection fraction (%)	41.5 ± 5.9	39.4 ± 11.7	n.s.
Change in ejection fraction (%)	5.3 ± 5.2	0.8 ± 8.4	0.046

**Figure 6:**
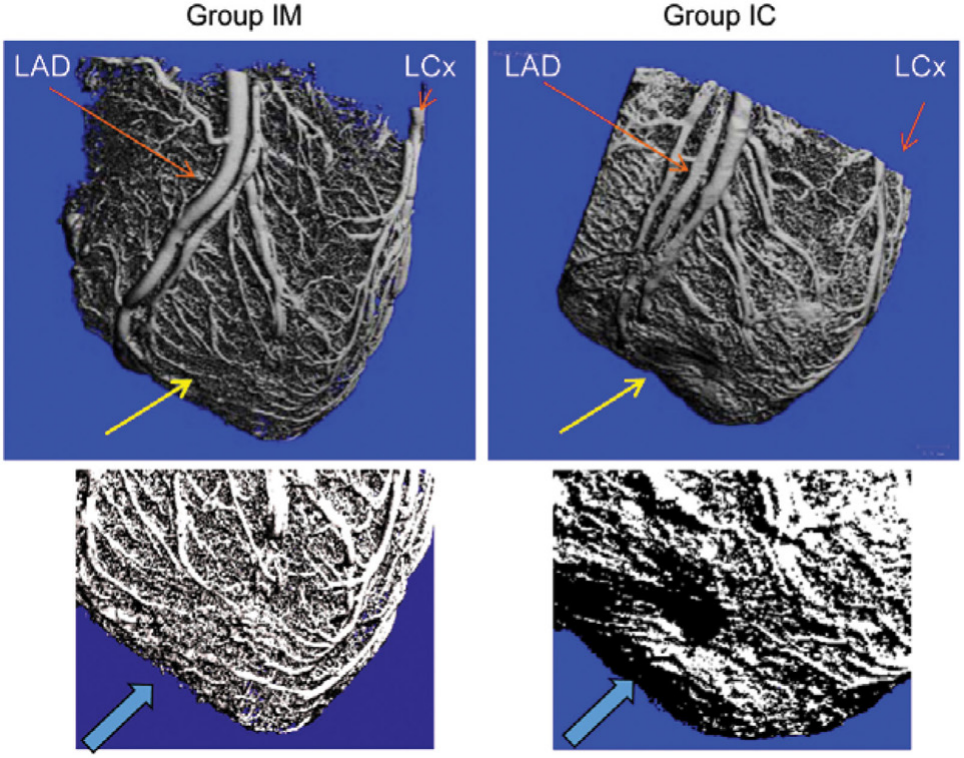
MicroCT of the infarcted and green fluorescent protein-Luc-mesenchymal stem cell (MSCs)-treated hearts. Microvascularization in the infarcted area (yellow arrow) 1 month after intramyocardial (group IM) or intracoronary (group IC) delivery of MSCs. Repeated micro CT image focusing on the anterior apical area of infarction (arrow).

MicroCT investigation of one heart of each group showed a higher capillary density in the infarcted area in group IM as compared to a heart of group IC (Figure 6).

## Discussion

Low coronary blood-flow triggered expression of myocardial MMP-2 followed by decrease of the chemotactic signals for cell homing, resulting in limited homing of injected cells, and lesser degree of angiogenic factor release, with consequent poorer 1-month outcome, as compared with intramyocardial delivery. We could demonstrate a direct relationship between diminished blood flow and increase in myocardial oxidative stress marker MMP-2, as well as a significant association between increase in MMP-2 and decrease of the homing factor CXCR4 in the cell-retention bioluminescence areas. Higher cell entrapment after intramyocardial cell delivery led to higher myocardial tissue levels of FGF2 and VEGF especially in that myocardial areas, where the cells were found.

### Retention of Cells After IC or Intramyocardial Delivery

In humans, two main delivery modalities have been utilized in cardiac stem cell clinical trials: IC infusion and intramyocardial delivery. Both routes have their advantages, and have shown moderate efficacy in cardiac regeneration therapy (Ben-Haim et al., 1996; Gepstein et al., 1997; Aarnoudse et al., 2007; Gyongyosi et al., 2008; DeCoux et al., 2014; Lukovic et al., 2016). Because intramyocardial cell transfer represents a more invasive procedure, IC cell delivery is more attractive for routine clinical use. However, despite the simplicity, acute ischemia due to stop-flow cell injection technique (repeated occlusion/reperfusion of the infarct-related artery) and the potential for intravascular cell clustering and distal embolization is a concern (Gyongyosi et al., 2010a, 2011). Our results are in line with the observations that higher myocardial accumulation of injected cells with consequent better efficacy profile could be demonstrated if the transplanted cells were injected intramyocardially (Collantes et al., 2017; Kanelidis et al., 2017). In addition to the previous works, we have revealed that intramyocardial injections led to less biodistribution in remote organs, and that intramyocardial injections resulted in higher expression of angiogenic substances in that myocardial areas, where the cells were injected and retained.

### Increased Oxidative Stress After IC Injection of Regenerative Cells

In accordance with the decreased blood flow and induced oxidative stress, myocardial expression of MMP-2 was increased in our experiment with diminished CXCR4-mediated homing signal in the IC group, with consequently diminished number of homed stem cells. Increased MMP-2 (both the 72 kD and its 64 kD activated form) expression has already been shown in injured carotid artery, which was remarkably enhanced and dominated by low-flow conditions (Bassiouny et al., 1998). In contrast with the carotid artery, where increased MMP-2 contributes to migration of smooth muscle cells to injury site, the increased expression of MMP-2 in the infarcted heart cleaves the SDF-1alpha/CXCR4 axis, resulting in accumulation of toxic proteolytic remnants, causing also reduced CXCR4-mediated homing signal, as our experiment showed (Giricz et al., 2006; Segers et al., 2007; Rota et al., 2008).

Homogenized myocardial tissue samples from the bioluminescence area (where the GFP-Luc-MSCs were found) showed higher expression of cadherin, SDF1alpha and CXCR4, as well as FGF2 and VEGF in the both groups, suggesting that the secretion of the homing and angiogeneic substances can be at least partly attributed to the injected GFP-Luc-MSCs. However, according to cross-talk of the transplanted stem and host myocardial cells, it is not possible to separate, which cells are responsible for the paracrine activity found in the myocardial areas, where the stem cells were retained.

Engrafted MSC secrete a variety of soluble trophic factors that mediate beneficial paracrine effects in the surrounding cells, especially proangiogenic factors (Bussche and Van de Walle, 2014) and, therefore, creating a favorable microenvironment. MSC may achieve protection by paracrine effects through released mediators rather than direct cardiac regeneration. In addition, less cardiomyocyte apoptosis (Nguyen et al., 2010) and attenuation of the myofibroblast transition in response to reduced oxygen and mechanical stress (Galie and Stegemann, 2014) greatly contribute to cardiac repair. Therefore, MSC and their secretome can reduce tissue injury, protect tissue from further adverse effects, and enhance tissue repair (Gnecchi et al., 2012; Gallina et al., 2015). Considering the limited homing and enhanced cell biodistribution after cell-based cardiac therapies, cell-free therapy injecting the paracrine factors released by stem cells *in vitro* came to foreground in the last years. However, up to now, neither single factor, nor combination of factors or survival cocktail could achieve similar reparative effect as did the stem cells.

Several investigators reported separate results on oxidative stress-induced MMP-2, or homing and biodistribution of injected cells or paracrine effect, to best of our knowledge, our study is the first to demonstrate the role of MMP-2 in cell homing, biodistribution, and paracrine effect of the retained cells, in its entire complexity. By using the *in vitro* bioluminescesce technology, we could localize macroscopically the injected living cells, and investigate the fingerprint of the cells on the myocardium and the additive influence of the local milieu on the injected cells, in terms of release of local paracrine factors.

### Imaging of the Cardiac Delivered Stem Cells

In order to investigate the direct effect of the cells in the tissue of our porcine model, we made the cells visible with *in vitro* bioluminescence imaging (Gyongyosi et al., 2010a, 2011). In accordance with the delivery technique, relatively high focal intensity signal could be seen after intramyocardial delivery, while obviously weaker signal was displayed post-IC delivery. Our results confirm previous radiolabeling studies showing poorer retention of stem cells after IC delivery compared to intramyocardial delivery (Hou et al., 2005). Similar to our results, Hale et al. have detected transplanted MSCs in the untreated myocardial areas 1 week after intravenous or intramyocardial delivery, which may reflect the movement of these cells from the infarct zone into the surrounding infarcted and the non-ischemic areas (Hale et al., 2008). We have also observed a widespread distribution of cells into non-target organs such as the liver, spleen, kidney, mediastinal lymph node, pericardium, and bone marrow with mild accumulation of the GFP-Luc-MSCs after 7 days mainly in liver, spleen, and bone marrow. With the exception of the lymph nodes, MSCs were found in organs of the reticuloendothelial system or the bone marrow, the latter being the expected site for “homing” of MSCs. Whether the decreased homing signal in the myocardium contributed to higher level of biodistribution of cells into remote organs remains to be elucidated.

### Limitations

We have not included normal, healthy animals as control group, because we intended to compare the differential effects of the two delivery modes on homing, paracrine function, and biodistribution of the injected cells. Additionally, we and other groups have already published data of control animals without MI and also MI without cell delivery, in comparison with cell-treated animals. Indeed, we have followed the 3R principles of the EU Commission (reduction, refinement and replacement) by reducing the number of the animals to the obligatory minimum. We have measured the myocardial MMP-2, CXCR4, cadherin, SDF-1alpha, FGF2, and VEGF also in the non-ischemic myocardium and compared the measured values to the ischemic infarcted and border areas. Even if it is known that the non-ischemic myocardium undergoes also certain biological processes [intrinsic remote ischemic conditioning by Pavo et al. (2017)], we assume that the expression levels of these factors in the remote areas may reflect the non-ischemic status, close to normal conditions.

We did not block MMP-2 activity by giving TIMP, since this was not the aim of our study. Preclinical studies have already been performed to investigate the cardioprotective effect of different MMP inhibitors, but unexpected systemic toxic effect has been observed by using TIMP (DeCoux et al., 2014): clinical development of most of the MMP inhibitors have been discontinued due to safety reasons (Dorman et al., 2007). Moreover, MMP-blocking in humans did not show beneficial effects on LV remodeling or clinical outcomes (Hudson et al., 2006). Constructing a specific MMP-2-inhibitor releasing system with biodegradable hydrogel for controlled release (CCT40CS), Fan et al. could overcome the pharmacological pitfalls of the MMP-2 inhibitors, namely the poor selectivity, toxicity, and fibrosis-inducing activity. Rats were subjected to anterior MI, and after randomization, animals of the respective groups received direct intramyocardial injections of CCT40CS 30 min post infarction. Results were compared with sham and infarction groups with no MMP-2 inhibitors. In these experiments, the authors have convincingly proven the role of MMP-2 in the infarcted rodent model, and the beneficial effects of a specific construct MMP-2 inhibitor on prevention of cardiac extracellular matrix degradation, cardiac remodeling, fibrosis and improvement of the cardiac function (Fan et al., 2017).

We have not counted the GFP-positive cells and the immunofluorecent stained cells because the quantification of the cell number in different areas of the heart and the remote organs are much more exact by using luciferase assay and ELISA from larger tissue samples (up to 5 mg) than the 4 µm thick limited size of microscopic pictures.

In conclusion, we have demonstrated IC stem cell delivery-associated decrease in myocardial blood flow and the consequent increase in myocardial MMP-2 and decreased CXCR4 expression with less cell homing and less angiogenic substance release. These led to diminished functional improvement of the infarcted left ventricle when compared with intramyocardial stem cell transfer, in the early phase of acute MI in pigs.

## Ethics Statement

All animal studies were approved by the local Experimental Animal Care Committee of University of Kaposvar, Hungary where the experiments were performed (EC 246/002/SOM2006, MAB-28-2005). The experiments conform to the “Position of the American Heart Association on Research.”

## Author Contributions

Planning of study: KZ, DL, RH, and MG. Conducting of experiments: all. Analysis: AG, JW, LM, DT, AS, SW, GZ, CK, AP, AG, ZP, ÖP, IR, RH-W, RM, FG, SC, KH, NP, IP, NN, and DK. Review: all.

## Conflict of Interest Statement

The research was conducted in the absence of any commercial or financial relationships that could be construed as a potential conflict of interest.

## Supplementary Material

Figure S1 Flow cytometry analysis of mesenchymal stem cells: cells are negative for CD 34 and CD 45 and positive for CD 29, CD 44, CD 90.

Figure S2 Cell proliferation assay post transfection of MSCs with Ad- green fluorescent protein (GFP) and Ad-Luc. Proliferation assay of the transfected and non-transfected porcine mesenchymal stem cells (MSCs). Transient transfection with adenovirus-associated luciferase and GFP resulted in a temporarily significant decrease in proliferation capacity.

Figure S3 Significant logarithmic correlation between marker matrix metalloproteinase-2 (MMP-2) 64 kD intensity and luciferase activity (index of green fluorescent protein-Luc-mesenchymal stem cell number) in the infarcted injected area.

## Abbreviations

AMF, absolute myocardial blood flow; CT, computer tomography; FACS, flow cytometry; FGF, fibroblast growth factor; FUP, follow-up; GFP, green fluorescent protein; IC, intracoronary; IM, intramyocardial; LAD, left anterior descending coronary artery; Luc, luciferase; LVEF, left ventricular ejection fraction; MI, myocardial infarction; MMP, matrix metalloproteinase; MRI, magnet resonance image; MSC, mesenchymal stem cell; SDF, stromal derived factor; VEGF, vascular endothelial growth factor.
