# Supplementary figures and images for: Matrix Metalloproteinase-2 Impairs Homing of Intracoronary Delivered Mesenchymal Stem Cells in a Porcine Reperfused Myocardial Infarction: Comparison With Intramyocardial Cell Delivery
Source: Front Bioeng Biotechnol. 2018 Apr 4;6:35. doi: 10.3389/fbioe.2018.00035 (PMC5893806; doi:10.3389/fbioe.2018.00035)

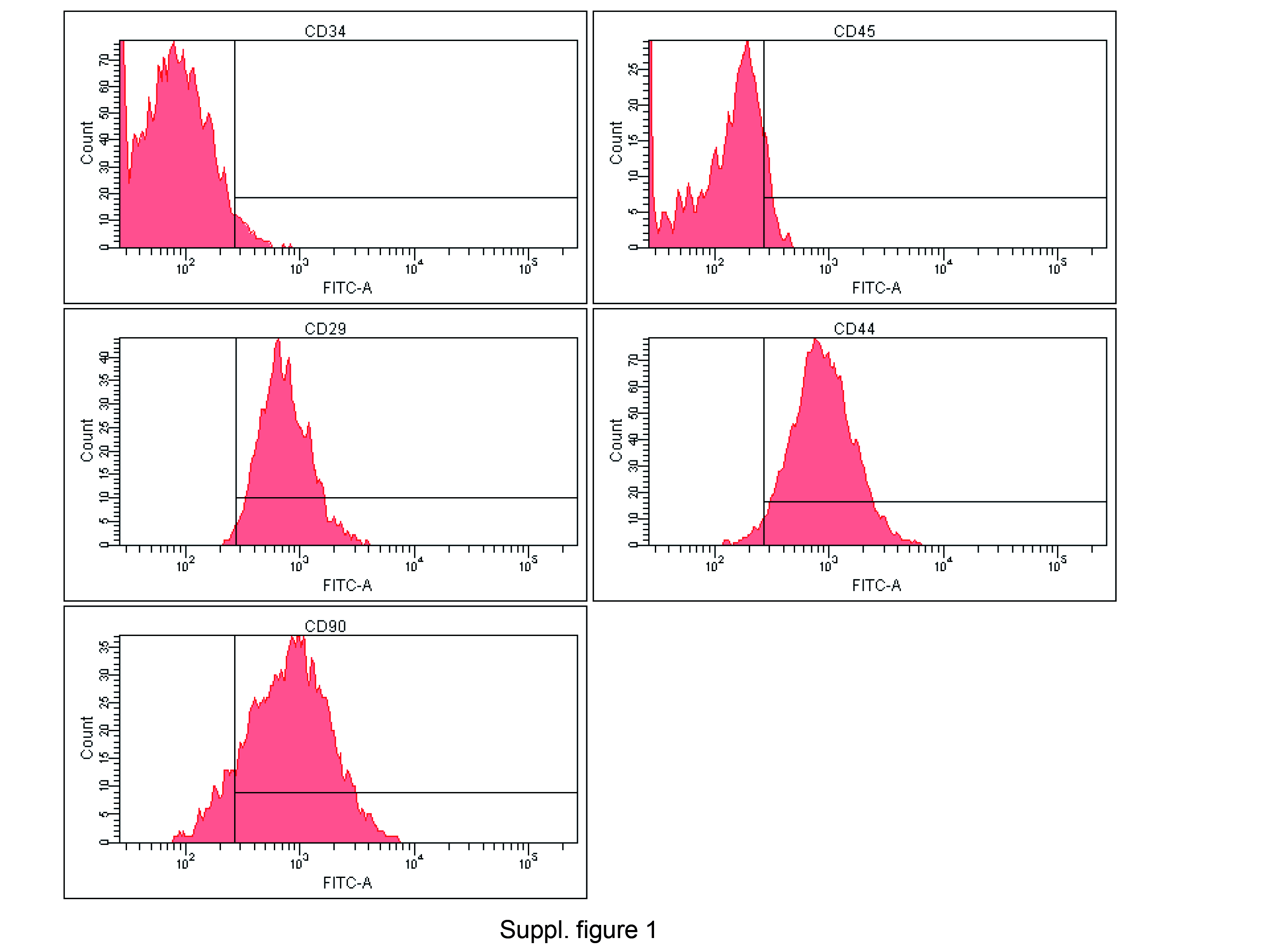

Supplement: Figure S1 — Flow cytometry analysis of mesenchymal stem cells: cells are negative for CD 34 and CD 45 and positive for CD 29, CD 44, CD 90. [file image_1.tif]

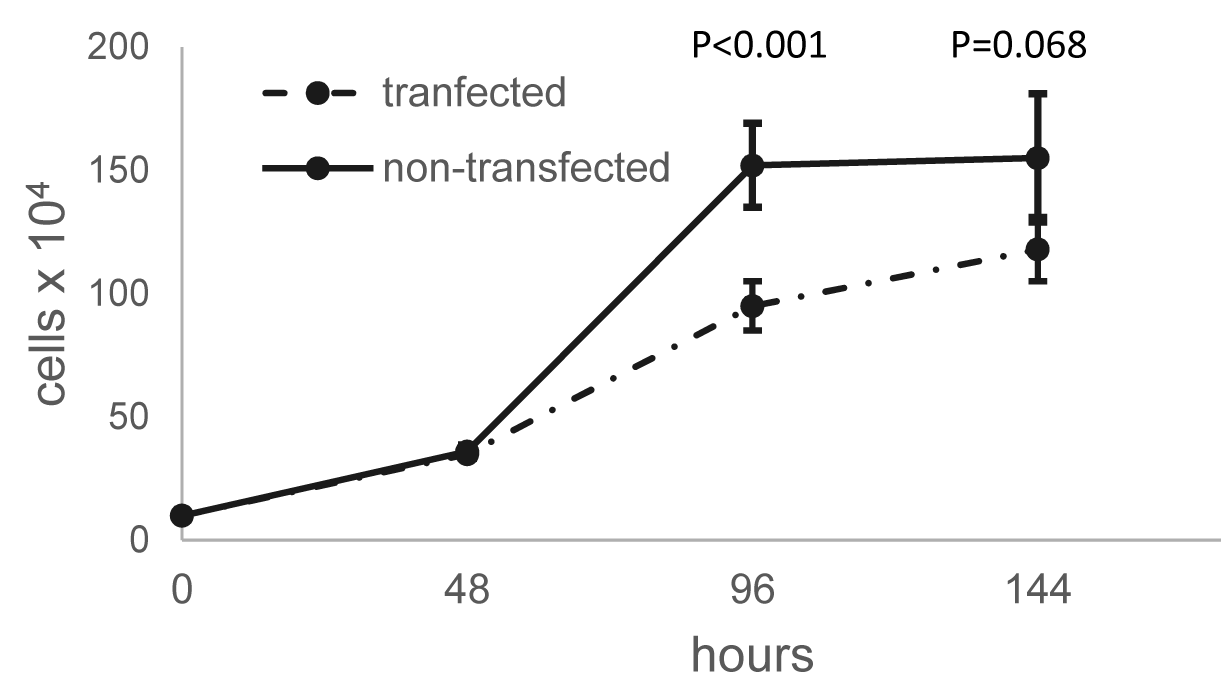

Supplement: Figure S2 — Cell proliferation assay post transfection of MSCs with Ad- green fluorescent protein (GFP) and Ad-Luc. Proliferation assay of the transfected and non-transfected porcine mesenchymal stem cells (MSCs). Transient transfection with adenovirus-associated luciferase and GFP resulted in a temporarily significant decrease in proliferation capacity. [file image_2.tif]

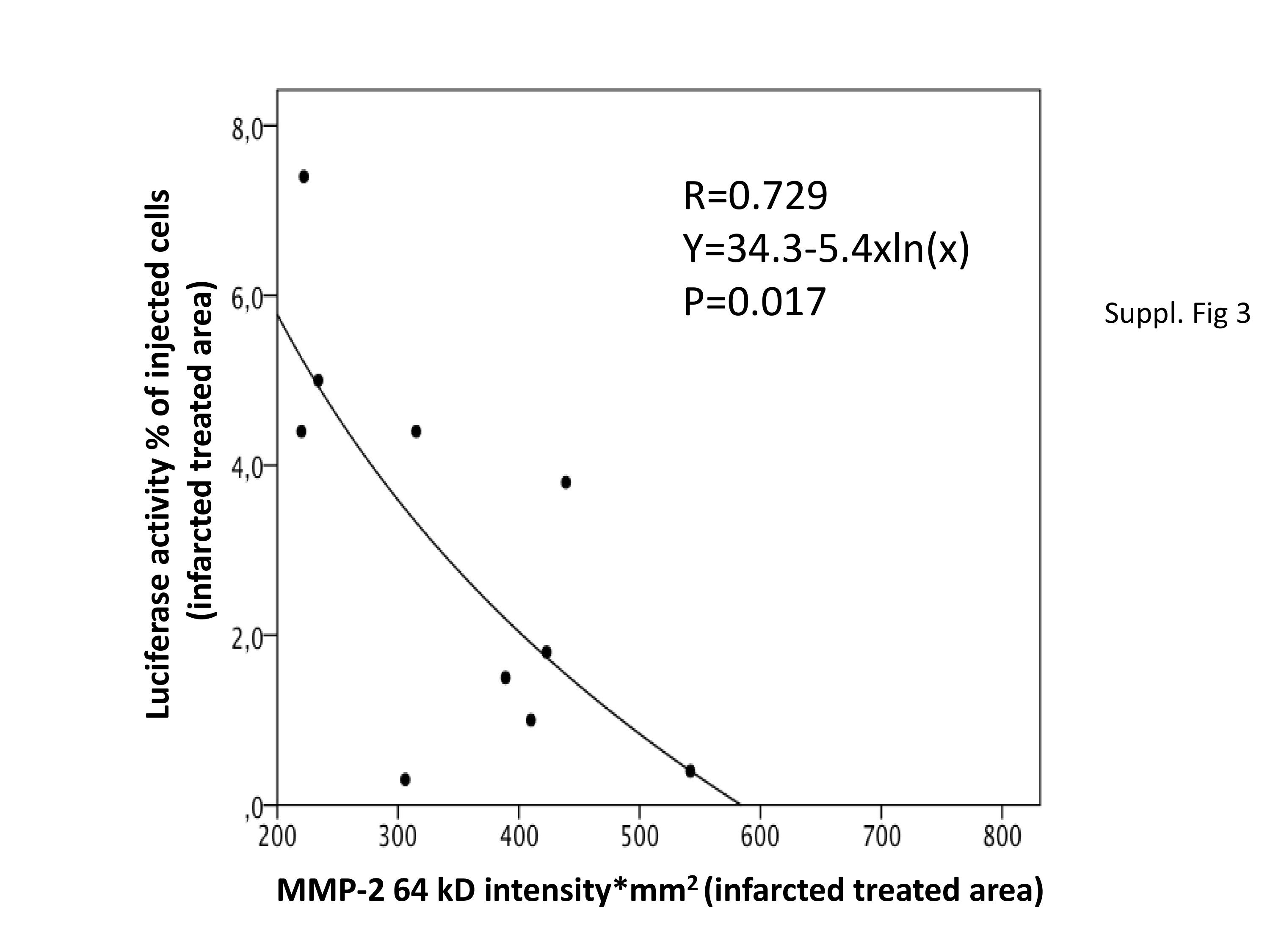

Supplement: Figure S3 — Significant logarithmic correlation between marker matrix metalloproteinase-2 (MMP-2) 64 kD intensity and luciferase activity (index of green fluorescent protein-Luc-mesenchymal stem cell number) in the infarcted injected area. [file image_3.tif]
